# Novel chimeric TLR2/NOD2 agonist CL429 exhibited significant radioprotective effects in mice

**DOI:** 10.1111/jcmm.16252

**Published:** 2021-02-19

**Authors:** Ying Cheng, Jicong Du, Ruling Liu, Suhe Dong, Jianming Cai, Fu Gao, Cong Liu

**Affiliations:** ^1^ Department of Radiation Medicine Faculty of Naval Medicine Naval Medical University Shanghai China

**Keywords:** Cl429, NOD2, radiation, radioprotection, TLR2

## Abstract

Severe ionizing radiation causes the acute lethal damage of haematopoietic system and gastrointestinal tract. Here, we found CL429, the novel chimeric TLR2/NOD2 agonist, exhibited significant radioprotective effects in mice. CL429 increased mice survival, protected mice against the lethal damage of haematopoietic system and gastrointestinal tract. CL429 was more effective than equivalent amounts of monospecific (TLR2 or NOD2) and combination (TLR2 + NOD2) of molecules in preventing radiation‐induced death. The radioprotection of CL429 was mainly mediated by activating TLR2 and partially activating NOD2. CL429‐induced radioprotection was largely dependent on the activation of TLR2‐MyD88‐NF‐κB signalling pathway. In conclusion, the data suggested that the co‐activation of TLR2 and NOD2 could induce significant synergistic radioprotective effects and CL429 might be a potential high‐efficiency selective agent.

## 1 INTRODUCTION

Acute radiation exposure is a serious public and military health problem.^1^ Severe ionizing radiation exposure causes the lethal damage of haematopoietic system and gastrointestinal tract, also known as acute radiation syndrome.^2^, ^3^ In addition, radiation‐induced damage is still the mainly dose‐limiting factor in radiotherapy.^4^

Toll‐like receptors (TLRs), as one of the major families of pattern recognize receptors (PRRs), play significant roles in immune system process and inflammatory response.^5^ In the year of 2008, Burdelya et al^6^ firstly reported that the activation of TLR5 exhibited strong radioprotective effects through activation of nuclear factor‐κB (NF‐κB). This discovery brought new insights into radioprotector development. The nucleotide‐binding oligomerization domain (NOD)‐like receptors (NLRs) are also crucial for host defence against bacterial infection.^7^, ^8^, ^9^ Stimulation of NOD1 and NOD2, two prototypic NLRs, could also trigger NF‐κB signalling pathway.^5^ In addition, the cross‐talk between TLRs and NLRs has been the highlight of many studies in recent years.^10^, ^11^, ^12^ For example, the functions of TLRs and NLRs brought new insights for vaccine development.^10^, ^13^, ^14^ Based on that, we try to address whether the activation of other kinds of PRRs could induce radioprotective effects, and whether the co‐activation of multiple PRRs could induce synergistic radioprotection.

CL429, composed of Murabutide (NOD2 ligand) covalently linked to Pam2C (TLR2 ligand) via a spacer, is a novel chimeric compound that was designed to activate both TLR2 and NOD2.^15^ It has been shown that CL429 induced synergistic immune function compared with combinations of separate ligands.^10^, ^15^, ^16^ In this study, we found that the co‐activation of TLR2 and NOD2 could induce strong synergistic radioprotective effects and CL429 might be a potentially highly effective and selective radioprotective agent.

## 2 MATERIALS AND METHODS

### 2.1 Chemicals and reagents

CL429 (Cat. Code: vac‐c429) was purchased from InvivoGen. Lipopolysaccharide (LPS) from *Escherichia coli* was purchased from Sigma (St.Louis, MO, USA), and Pam3CSK4 was obtained from EMC Microcollection (Tübingen, Germany). The TransDetect Annexin V‐FITC/PI Cell Apoptosis Detection Kit was purchased from TransGen Biotech (Beijing, China). RPMI 1640 and foetal bovine serum (FBS) were supplied by Gibco. Streptavidin FITC, anti–Mouse CD117 APC eFluor 780, and anti‐Mouse Ly‐6A/E (Sca‐1) PerCP‐Cyanine5.5 were purchased from BD Pharmingen (San Diego, CA). Lineage Cell Detection Cocktail‐Biotin was purchased from Miltenyi Biotec (Germany). Lipofectamine 3000 was purchased from Thermo Fisher. Antibodies were purchased from Affinity Biosciences (Jiangsu, China) and Cell Signaling Technology (Massachusetts, USA). Commercial ELISA kits were purchased from DAKEWE (Beijing, China).

### 2.2 Animals and treatment

Wild‐type male, aged 6‐8 weeks C57BL/6 mice were purchased from China Academy of Science (Shanghai, China). TLR2 KO mice were purchased from Model Animal Research Center, Nanjing University. All mice were housed in laboratory animal room under standard conditions. The experiments were approved by the Laboratory Animal Center of the Naval Medical University, China, in conformance with the National Institute of Health Guide for the Care and Use of Laboratory Animals. Mice were treated with CL429 (2.5 mg/kg), LPS (2.5 mg/kg), WR2721 (150 mg/kg), MDP (5 mg/kg), and Pam3CSK4 (50 ng/mg) via peritoneal injection 24 and 2 hours before total body irradiation (TBI). The mice in control group treated with phosphate buffered saline (PBS). ^60^Co source in the radiation centre (Faculty of Naval Medicine, Naval Medical University, China) was used to irradiate mice and cells.

### 2.3 Cell culture and treatment

Human intestinal epithelial cell (HIEC) was obtained from American Type Culture Collection and cultured in RPMI 1640 with 10% FBS at 37°C in a 5% CO_2_ humidified chamber.

### 2.4 siRNA transfection

TLR2 siRNA (si‐TLR2_001 CCAATCTCACAAATTTACA, si‐TLR2_002 CATTTGGATTTGTCTGATA, si‐TLR2_003 CAACAATCTTGACTCATTT) and NOD2 siRNA (si‐Nod2_002 GCAACAGCGTGGGTGATAA, si‐Nod2_003 GCACAGAGTTGCAACTGAA, siNod2_004 GCGAGCACTTCCATTCCAT), purchase from Guangzhou RiboBio (Guangzhou, China), were used to knock down TLR2 or/and NOD2 in HIEC cells. Lipofectamine 3000 was used to transfect siRNA according to the protocol. Western blot was used to verify gene expression efficacy. At 72 hours after siRNA transfection, cells were treated with CL429 (40 µg/mL) 12 and 2 hours before irradiation. At 24 hours after irradiation, cell viability was determined using CCK‐8.

### 2.5 The relative number of bone marrow cells (BMCs)

The relative number of BMCs was determined by flow cytometry (Beckman Cytoflex). The femur of mice was washed repeatedly with 1 mL PBS for 3 times. Then, the cell suspension was centrifuged at 1000 *g* for 5 minutes. After the supernatant discarded, the pellet was lysed with 1 mL Red Blood Cell Lysis Buffer for 10 minutes at 4℃ to remove the RBCs. The leaving BMCs were washed and resuspended with 1 mL PBS. Flow cytometry was then performed to enumerate the BMCs of each femur within 1 minute at the sample flow rate of 15 μL/min.

### 2.6 Antibody staining and flow cytometry

The cell apoptosis was analysed using the apoptosis detection kit. After radiation, bone marrow cells (BMCs) were isolated freshly. Then, cells were strained through a 40‐μm strainer in the presence of PBS and red blood cells were removed. Cells were stained with antibody for 20 minutes at 4°C. TenF thousand cells were analysed by flow cytometry (Beckman Cytoflex) in accordance with the manufacturer's instructions.

### 2.7 Histological examination

Mice tissues were removed and then fixed in 4% paraformaldehyde after irradiation. After embedded in paraffin, the tissues were stained with haematoxylin and eosin (HE). The relative length of villus was measured using ImageJ software (National Institutes of Health, USA).

### 2.8 TUNEL assay and Ki67 assay

Mice small intestinal tissues were removed and then fixed in 4% paraformaldehyde at 24 hours post‐irradiation. TUNEL (Terminal deoxynucleotidyl transferase dUTP nick‐end labelling) staining and Ki67 assay were did according to the manufacturer's instructions. The TUNEL+ cells were counted in 10 crypts per section. The Ki67‐positive area per section was measured using ImageJ software (National Institutes of Health, USA).

### 2.9 Enzyme‐linked immunosorbent assay (ELISA)

Blood was collected from the mice 24 hours post‐irradiation and stored at room temperature for 30 minutes. Then, the blood samples were centrifuged at 4000 *g* for 10 minutes. Serum was collected and stored at −20°C. The levels of IL‐6, IL‐11, IL‐12 and TNF‐α in serum were analysed by ELISA according to the manufacturer's instructions. The OD value was measured at 450 nm using Multi‐Mode Reader (BioTek, Vermont, USA) and calculated at the linear portion of the curve.

### 2.10 Statistical analysis

Data were expressed as mean ± SD. Two‐tailed Student's *t* test was used to analyse the difference between 2 groups. One‐way ANOVA was employed to analyse the difference among 3 groups. Kaplan–Meier analysis was applied to estimate the difference of overall survival. The data were analysed using SPSS ver. 19 (IBM Corp, Armonk, NY, USA). *P* < .05 was considered statistically significant.

## 3 RESULTS

### 3.1 CL429 exhibited significant radioprotective effects in vivo

**FIGURE 1:**
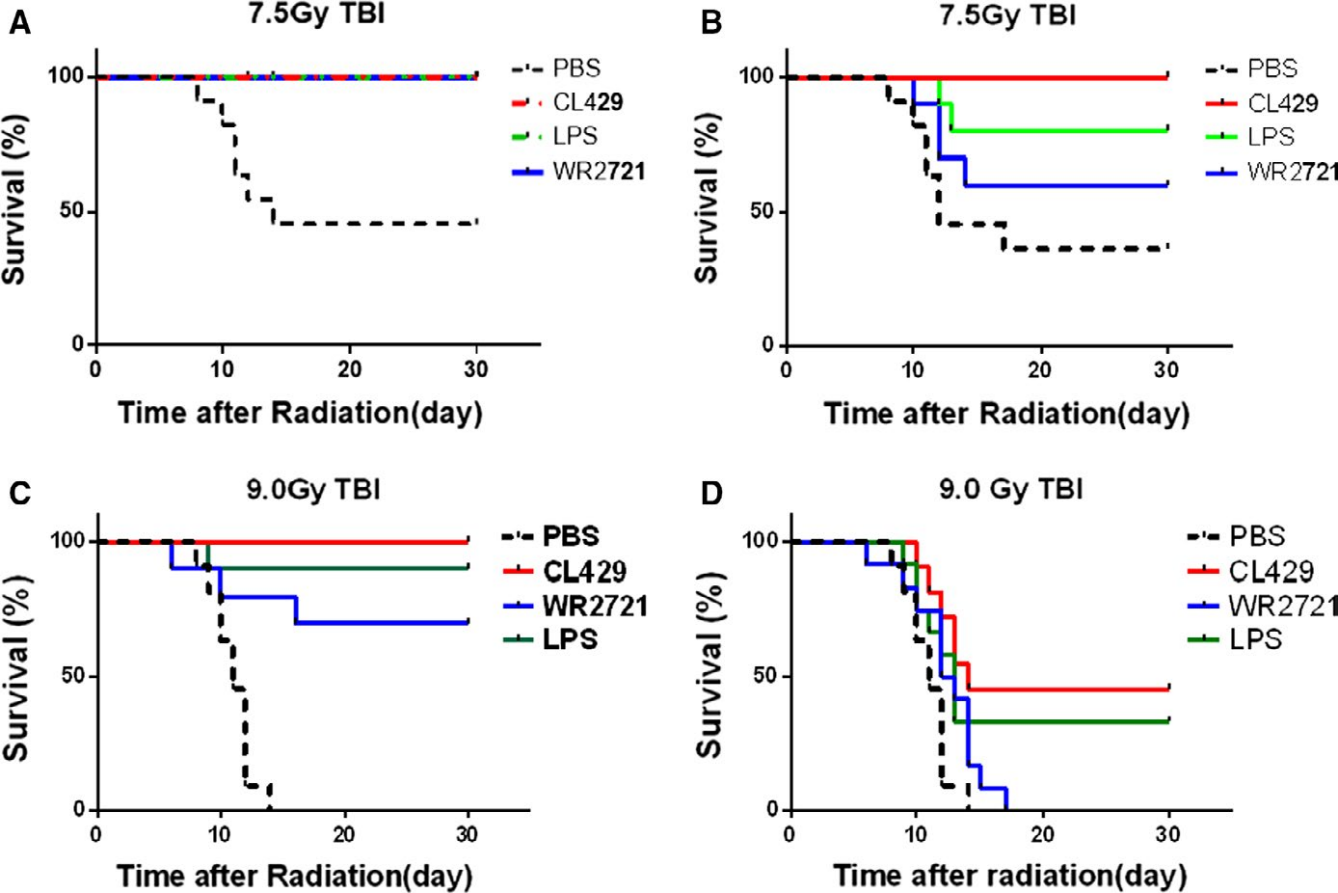
CL429 exhibited significant radioprotective effects in vivo. (A/C) C57BL/6 mice (n = 10) were pre‐treated with PBS, CL429 (5 mg/kg), LPS (2.5 mg/kg) and WR2721 (150 mg/kg) via peritoneal injection 24 and 2 h before 7.5 Gy or 9.0 Gy TBI. Then, the survival was recorded. (B/D) C57BL/6 mice were administered with PBS, CL429 (5 mg/kg), LPS (2.5 mg/kg) and WR2721 (150 mg/kg) via peritoneal injection immediately after 7.5 Gy or 9.0 Gy TBI. The survival was recorded

To determine the radioprotective effects of CL429 in vivo, the survival of C57BL/6 mice was recorded after 7.5 Gy or 9.0 Gy TBI. 100% of mice in the CL429 pre‐treated group survived 30 days, while all PBS pre‐treated mice died in 14 days after 9.0 Gy TBI. In addition, lipopolysaccharide (LPS) prevented 90% of mice radiation‐induced death. TLR5 agonist and WR2721 also exhibited radioprotective effects after 9.0 Gy TBI (Figure 1C). Furthermore, administrated with CL429 immediately after 9.0 Gy TBI, 70% of mice survived for at least 30 days. In contrast, the survival rates of other groups were lower than 40% (Figure 1D). CL429 also showed great radioprotective effects at the dose of 7.5 Gy TBI (Figure 1A/B). Taken together, CL429 exhibited significant radioprotective effects in vivo.

### 3.2 CL429 protected mice against lethal radiation‐induced haematopoietic system injury

**FIGURE 2:**
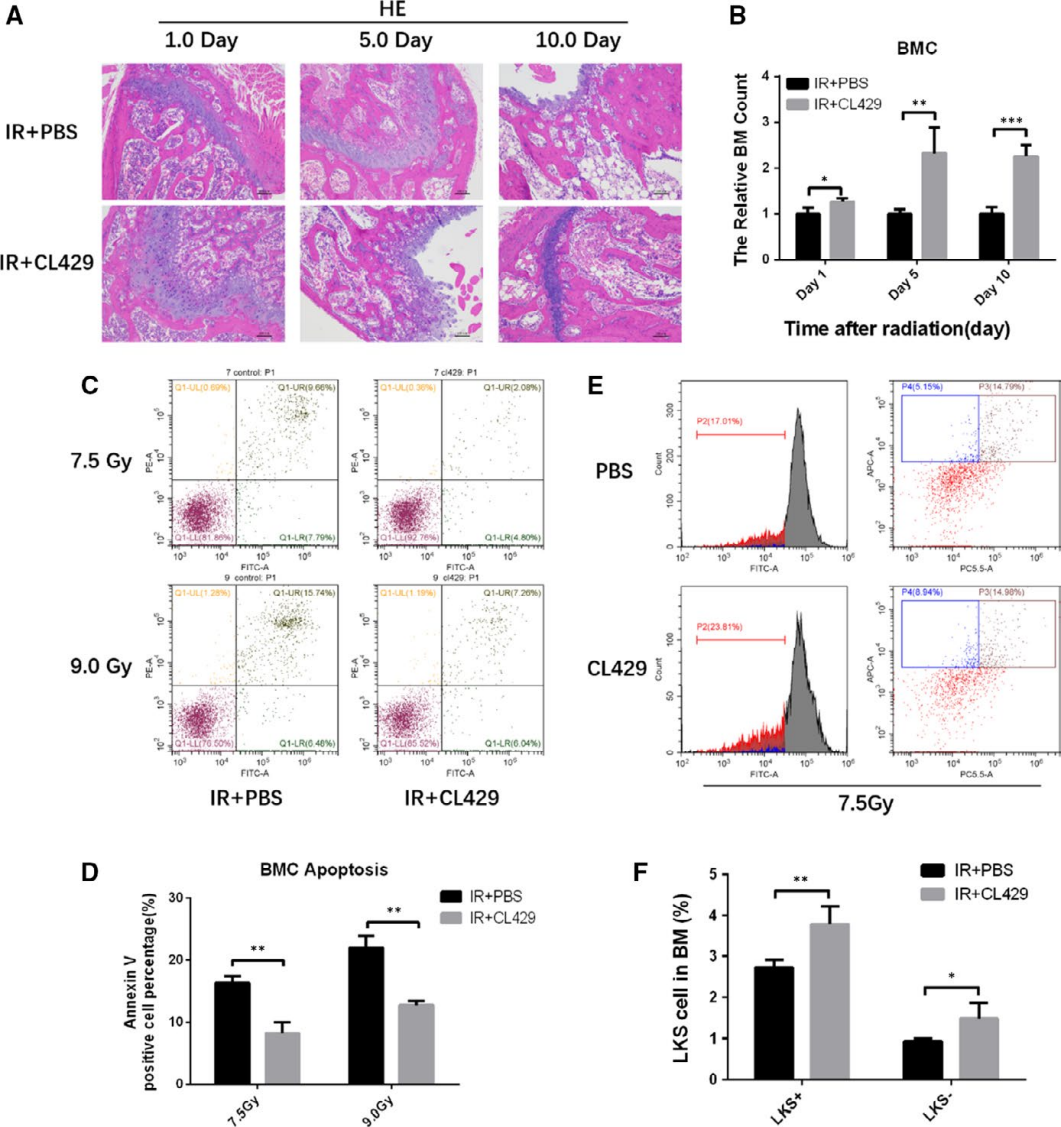
CL429 protected mice against lethal radiation‐induced haematopoietic system injury. C57BL/6 mice were treated with PBS or CL429 before 7.5 Gy TBI. (A) Representative images of HE‐stained bone marrow sections with the indicated treatment at 1, 5 and 10 d after 7.5 Gy TBI. (B) The number of BMCs was determined at 1, 5 and 10 d after 7.5 Gy TBI. (C) BMCs were isolated from mice 24 h after radiation; then, the apoptosis of BMCs was analysed by flow cytometry. (D) The data of apoptosis were presented as mean ± SD. (E) The number of LKS+ and LKS‐ cells were analysed by flow cytometry 24 h after 7.5 Gy radiation. (F) The data of LSK cells were presented as mean ± SD. *P < .05 and **P < .01 for control vs CL429 treatment

HE staining was used to estimate the radioprotection of CL429 on the haematopoietic system. The pathology findings showed that CL429 group had better‐preserved haematopoietic system structure and recovered faster than the PBS group after 7.5 Gy TBI (Figure 2A). In addition, the relative number of BMCs in CL429 group was also higher than PBS group (Figure 2B). Next, the apoptosis of BMCs was detected using flow cytometry, and we found that the apoptosis rate was decreased significantly in CL429 group compared to PBS group (Figure 2C/D). Haematopoietic stem cells (HSCs) play critical roles in reconstitution of haematopoietic system after radiation. By using flow cytometer, we found that CL429 statistically increased the number of LSK cells after 7.5 Gy TBI (Figure 2E/F). Those data demonstrated that CL429 could alleviate radiation‐induced haematopoietic system injury and promote it repair by inhibiting BMCs apoptosis and up‐regulating the number of LSK cells.

### 3.3 CL429 prevented lethal radiation‐induced intestinal injury

**FIGURE 3:**
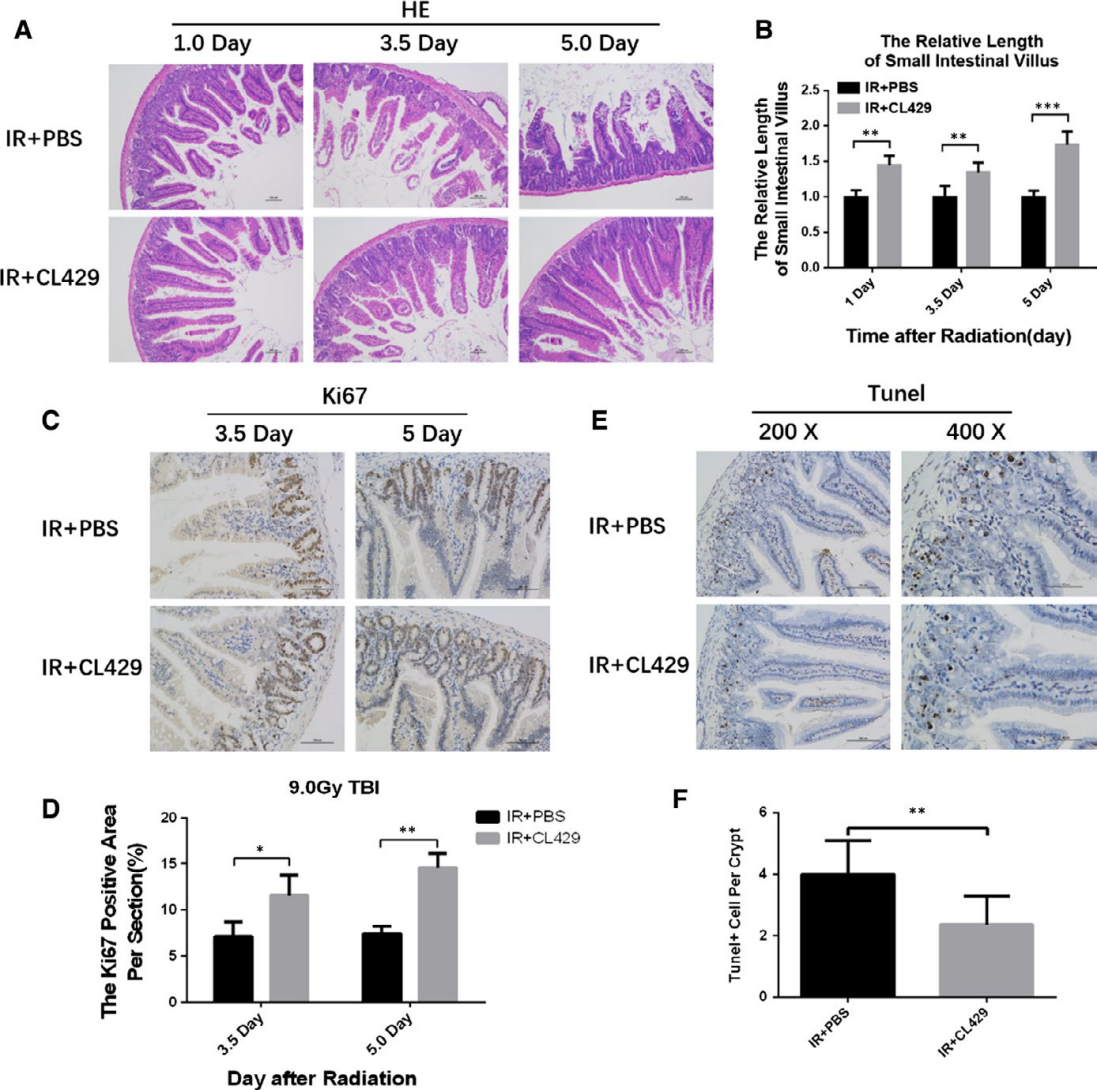
CL429 prevented lethal radiation‐induced intestinal injury. C57BL/6 mice were pre‐treated with PBS or CL429 before 9.0 Gy TBI. (A) Representative images of HE‐stained intestinal sections with the indicated treatment at 1.0, 3.5 and 5.0 d after TBI. Scale bar: 100 μm. (B) The relative length of small intestine villus at 96 h after radiation. (C) The representative images of Ki67‐stained intestinal sections. Scale bar: 100 μm. (D) The quantification of Ki67 positive area per section. (E) The representative images of TUNEL‐stained intestinal sections. Scale bar: 100 μm. (F) The quantification of TUNEL^+^ cells per crypt. *P < .05 and **P < .01 for control vs CL429 treatment

Gastrointestinal tract is another tissue which is sensitive to ionizing radiation. Using HE staining, we found that CL429 group exhibited greater intestinal structure, taller villi, and more surviving crypts (Figure 3A/B). To understand the radioprotective effects of CL429 on crypts regeneration, Ki67 staining was used to evaluate the proliferation of crypts, and we found that crypts proliferation was decreased significantly in the PBS group compared with the CL429 group 3.5 days after TBI. The crypts proliferation of CL429 was reaching almost twice that of the PBS group 5.0 days after TBI (Figure 3C/D). Then, we detected crypts apoptosis using TUNEL assay. The results showed that the number of TUNEL^+^ cells per crypt was decreased in CL429 group compared to PBS group (Figure 3E/F). Those data proved that CL429 could protect mice against radiation‐induced intestinal injury by promoting crypts proliferation and suppressing crypts apoptosis.

### 3.4 CL429 induced synergistic radioprotective effects compared with the combination of separate ligands

**FIGURE 4:**
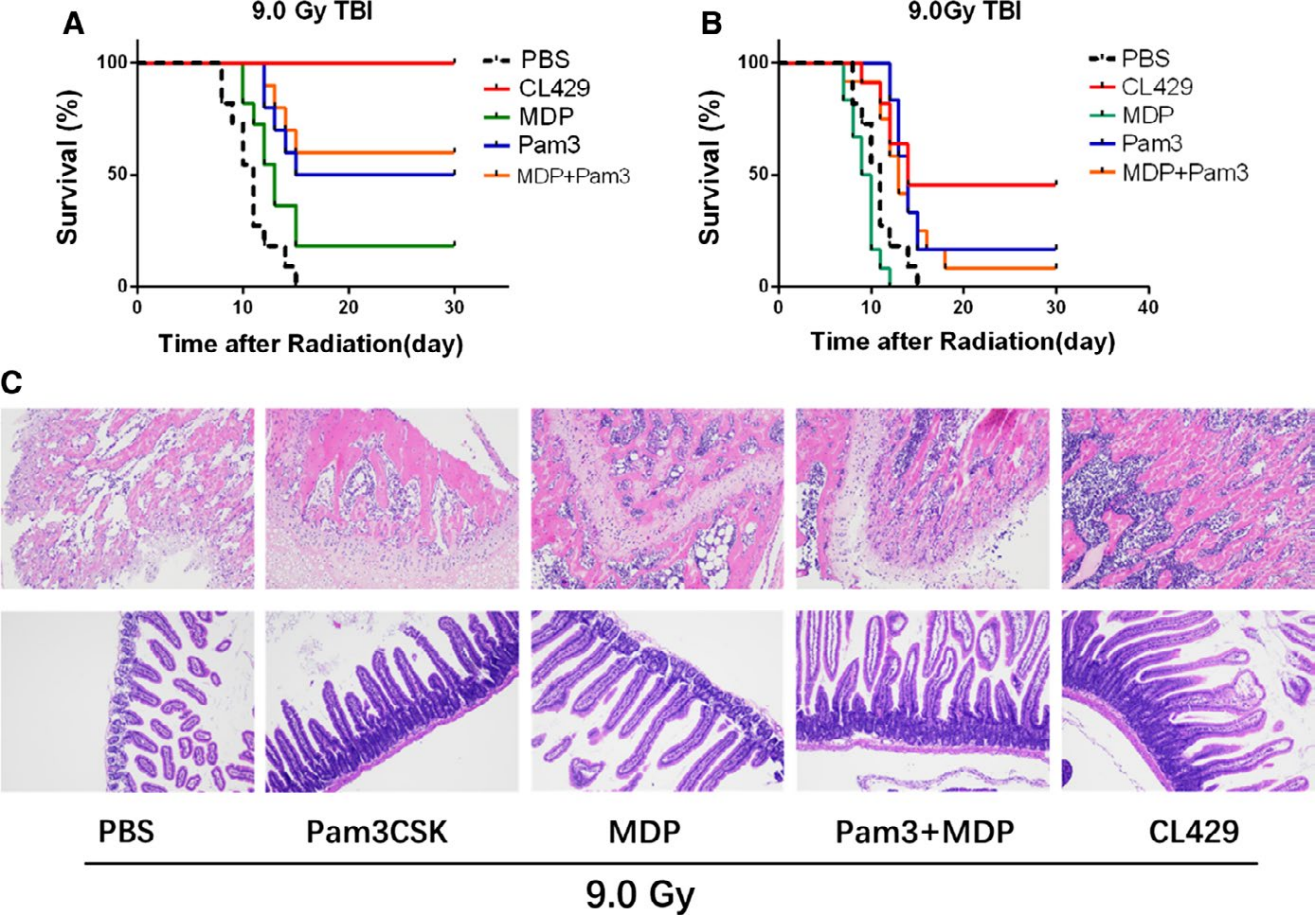
CL429 induced synergistic radioprotective effects compared with the combination of separate ligands. (A) C57BL/6 mice were pre‐treated with CL429, Pam3CSK (TLR2 ligand), MDP (NOD2 ligand), and Pam3CSK + MDP before TBI, and then, the survival was monitored. (B) C57BL/6 mice were treated with CL429, Pam3CSK (TLR2 ligand), MDP (NOD2 ligand), and Pam3CSK + MDP immediately after TBI, and then, the survival was monitored. (C) The representative pathological images of haematopoietic system and gastrointestinal tract at 3.5 d after radiation

As a chimeric TLR2/NOD2 agonist, CL429 stimulates both TLR2 and NOD2 and induce NF‐κB and/or AP‐1 signing pathway. To determine the radioprotective effects of TLR2 and/or NOD2 ligands in vivo, mice were treated with CL429, Pam3CSK4 (TLR2 ligand), MDP (NOD2 ligand) and Pam3CSK4 + MDP, respectively; then, the survival rates of mice were monitored after 9.0 Gy TBI. As showed in Figure 4A, all CL429‐treated mice survived over 30 days, while 60% of mice treated with Pam3CSK4 + MDP survived over 30 days. In addition, Pam3CSK prevents 50% of mice radiation‐induced death, and MDP only protected 20% mice against radiation‐induced death. The pathological results of haematopoietic system and gastrointestinal tract were also consistent with the survival results (Figure 4C). Furthermore, the survival of mice administrated with those agonists immediately after 9.0 Gy TBI showed that CL429 was more effective than Pam3CSK4 + MDP and Pam3CSK4, while MDP had no therapeutic effect (Figure 4B). Taken together, those data indicate that CL429 was more effective than equivalent amounts of monospecific (TLR2 or NOD2) and combination (TLR2 + NOD2) of molecules in preventing radiation‐induced injury. The chimeric TLR2/NOD2 ligand CL429 induced synergistic radioprotective effects compared with the combination of separate ligands. In addition, those results also reminded us that the activation of NOD2 might have radioprotective effects.

### 3.5 CL429‐induced radioprotective effects were mainly mediated by TLR2 and partially mediated by NOD2

**FIGURE 5:**
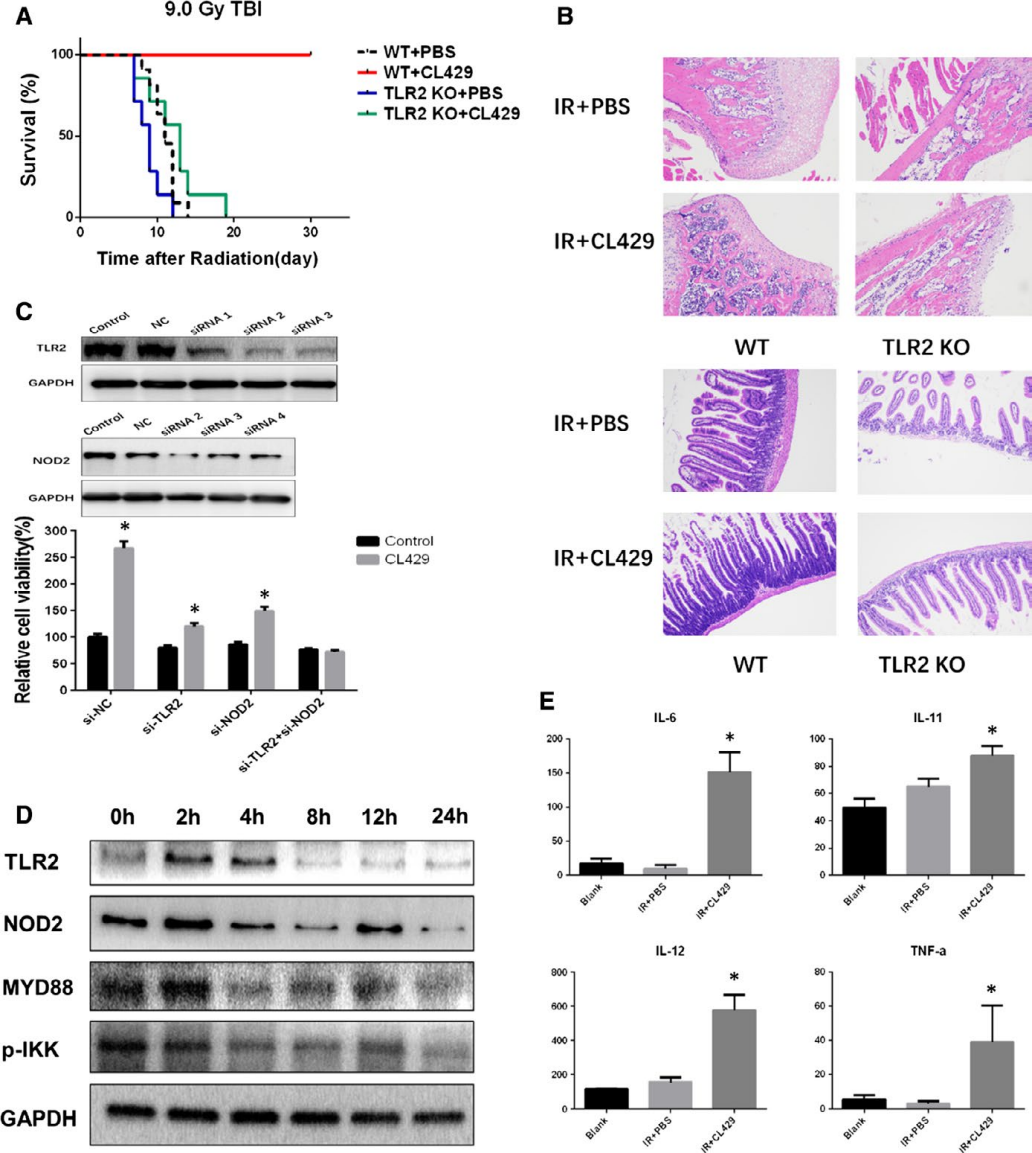
The mechanism for the radioprotective effect of CL429. (A) C57BL/6 mice and TLR2 KO mice were treated with CL429 before TBI, and then, the survival was monitored (WT n = 11, TLR2 KO n = 7). (B) The representative pathological images of haematopoietic system and gastrointestinal tract at 3.5 d after radiation. (C) The protein expression of TLR2 and NOD2 after siRNA knock‐down. Cell viability was determined using CCK‐8 at 24 h after radiation. *P < .05 vs IR groups. (D) The effects of CL429 on TLR2, NOD2, MyD88 and p‐IKK at 0 h, 2 h, 4 h, 8 h, 12 h and 24 h were measured by Western blot assay. (E) IL‐6, IL‐11, IL‐12 and TNF‐α were detected by ELISA 24 h after radiation. *P < .05, **P < .01 vs IR + PBS groups

It has been proven that the chimeric ligand CL429 activated both TLR2 and NOD2. Firstly, we used TLR2 KO mice to clarify the potential radioprotective mechanism of CL429. As shown in Figure 5A, CL429 protected wild‐type mice from radiation‐induced death but had no radioprotective effects on the TLR2 KO mice. The pathological results were also consistent with the survival results, which means most of radioprotective effects of CL429 were abrogated in TLR2 KO mice (Figure 5B). Next, TLR2 and NOD2 KD cell lines were established by siRNA transfection and Western blot was used to verify gene expression efficacy. TLR2 siRNA3 and NOD2 siRNA2 sequences were effective to knock down these receptors (Figure 5C). By using TLR2 and NOD2 KD cell lines, we found that the relative cell viability of NOD2 KD cells was higher than it of TLR2 KD cells, and both of their viability were lower than NC cells (Figure 5C).

And through Western blot, we observed increased expressions of TLR2, NOD2 and MyD88 after CL429 treatment and decreased expression of p‐IKK, indicating CL429 effectively activated TLR2‐MYD88‐NF‐κB signalling pathway (Figure 5D). Recent studies showed that the radioprotection of activating TLRs is accompanied by secreting of cytokines including IL‐6, IL‐11, IL‐12, TNF‐α and so on. These cytokines are of fundamental for radioprotection. By using ELISA, we found that IL‐6, IL‐11, IL‐12 and TNF‐α were significantly increased in CL429‐treated group in comparison with PBS group (Figure 5E). These data demonstrated that CL429‐induced radioprotective effects were mainly mediated by activating TLR2 and partially activating NOD2. And TLR2‐MYD88‐NF‐κB signalling pathway may play critical roles in CL429 induced radioprotection.

## 4 DISCUSSION

Acute severe radiation‐induced damage has been a worldwide problem.^17^, ^18^ Toll‐like receptors (TLRs) play essential roles in recognizing specific components of pathogenic microorganisms and triggering immune system responses.^19^, ^20^

In this study, we demonstrated that CL429, which can stimulate both TLR2 and NOD2, exhibited significant radioprotective effects in vivo. The radioprotective effects of CL429 were much better than WR2721 and LPS. Haematopoietic system is one of the most radiosensitive tissue. Firstly, we assessed the radioprotective effects of CL429 by using HE staining and flow cytometry. CL429 could alleviate radiation‐induced haematopoietic system injury and promote the reconstitution of haematopoietic system by inhibiting BMC apoptosis and up‐regulating the number of LSK cells. Next, we evaluated the intestinal radioprotection of CL429. As shown in Figure 3, the structure of intestine in CL429 group had greater intestinal structure, taller villi, more surviving crypts and less TUNEL^+^ cells in crypts. Those data proved that CL429 protected mice against radiation‐induced intestinal injury by promoting crypts proliferation and suppressing crypts apoptosis.

To figure out whether CL429 could induce synergistic radioprotective effects between TLR2 and NOD2 in vivo, we monitored the survival of mice treated with CL429, Pam3CSK4 (TLR2 ligand), MDP (NOD2 ligand), and Pam3CSK4 + MDP before TBI. The survival data suggested that CL429 was significantly more effective than equivalent amounts of monospecific (TLR2 or NOD2) and combination (TLR2 + NOD2) of molecules in preventing radiation‐induced injury. Compared to the combination of individual ligands, CL429 induced synergistic radioprotective effects. In addition, those results also reminded us that the activation of NOD2 might have radioprotection. KO mice and KD cells were used to clarify the potential mechanism of CL429. And we demonstrated that the radioprotection of CL429 was mainly mediated by activating TLR2 and partially activating NOD2. Next, we detected the expression levels of TLRs and NODs signalling pathways in HIEC after CL429 stimulation through Western blot, we observed increased expressions of TLR2, NOD2 and MyD88 after CL429 treatment and decreased expression of p‐IKK, indicating CL429 effectively activated TLR2‐MYD88‐NF‐κB signalling pathway. Moreover, we found IL‐6, IL‐11, IL‐12 and TNF‐α were significantly increased in CL429‐treated group in comparison with PBS group by using ELISA.

These data demonstrated that CL429‐induced radioprotective effects were mainly mediated by activating TLR2 and partially activating NOD2. And TLR2‐MyD88‐NF‐κB signalling pathway may play critical roles in CL429 induced radioprotection.

In recent years, TLRs has bought new development directions to radioprotection.^7^, ^8^, ^21^, ^22^ However, the agonist of TLR4, such as LPS, might have great radioprotection with high toxicity, whereas the agonist of TLR2 has lower toxicity with weaker radioprotection. To solve this problem, we found CL429, a novel chimeric compound designed to stimulate TLR2 and NOD2. Many studies have demonstrated that CL429 could induce synergistic immune function. And in this study, our data showed that CL429 induced synergistic radioprotective effects. Moreover, by using KO mice and KD cell lines, we illustrated that the radioprotective effects of CL429 were mainly mediated by TLR2 and partially mediated by NOD2.

The co‐activation of TLR2 and NOD2 could activate both NF‐κB and AP1 signalling pathways.^10^, ^16^ The chimeric ligands were significantly more effective than separated ligands. However, we still do not know where and how the synergistic radioprotective effects occur. It was found that some molecules may play key roles in the synergy, such as TAK1. As a ser/thr kinase, TAK1 participates in the TLR2 and NOD2 signalling pathways, resulting in proinflammatory cytokine secretion.^23^, ^24^, ^25^ This double activation could be part of the synergistic mechanism between these two receptors, but this potential mechanism has not been proven in radioprotective field. Moreover, we try to find new potential key targets by using the RNA sequencing technology.

## 5 CONCLUSION

**FIGURE 6:**
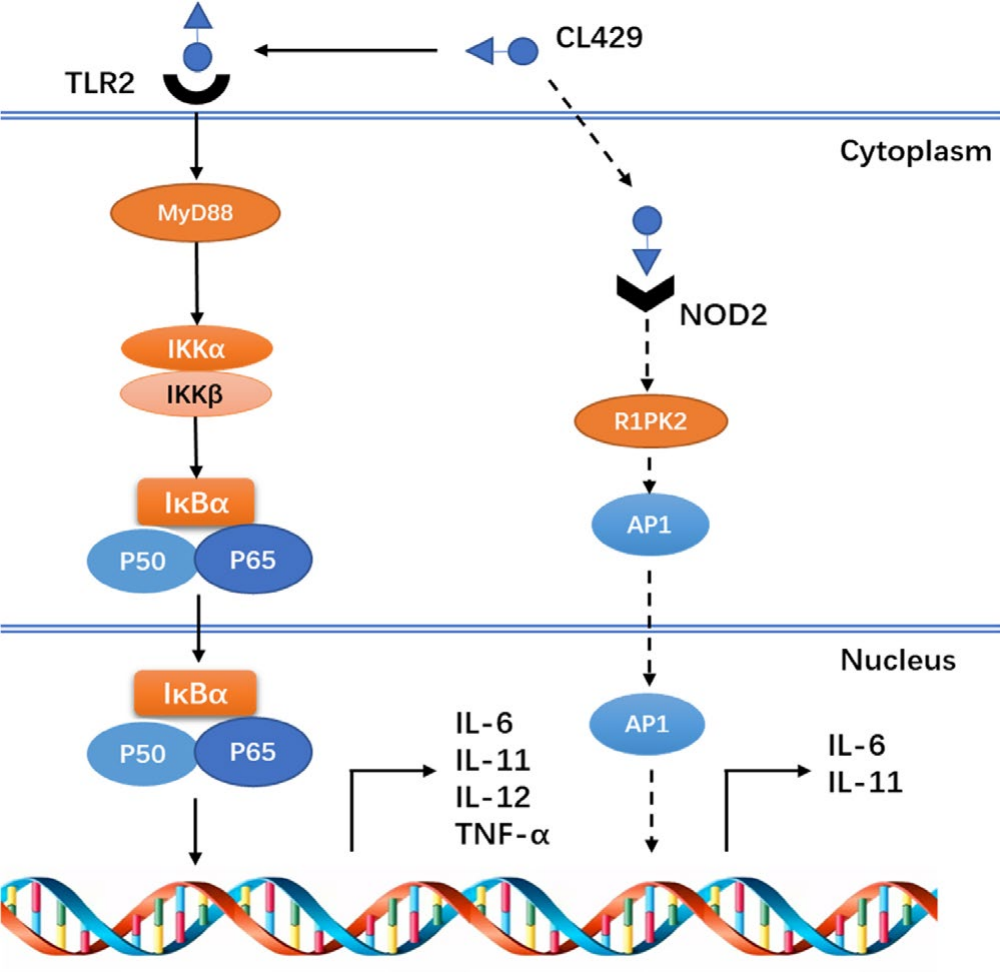
Possible mechanism of CL429 radioprotection

In conclusion, our data suggested that the co‐activation of TLR2 and NOD2 could induce significant synergistic radioprotective effects and CL429 might be a potentially highly effective and selective radioprotector (Figure 6).

## CONFLICT OF INTEREST

The authors confirm that there are no conflicts of interest.

## AUTHOR CONTRIBUTIONS

**Ying Cheng:** Investigation (equal); methodology (equal). **Jicong Du:** Writing‐original draft (equal). **Ruling Liu:** Formal analysis (supporting). **Suhe Dong:** Methodology (supporting). **Jianming Cai:** Resources (supporting). **Fu Gao:** Resources (supporting); writing‐review & editing (supporting). **Cong Liu:** Writing‐review & editing (supporting).

## Data Availability

The data that support the findings of this study are openly available.
